# T-cell exhaustion in immune-mediated inflammatory diseases: New implications for immunotherapy

**DOI:** 10.3389/fimmu.2022.977394

**Published:** 2022-09-23

**Authors:** Zhanyan Gao, Yang Feng, Jinhua Xu, Jun Liang

**Affiliations:** ^1^ Department of Dermatology, Huashan Hospital, Fudan University, Shanghai, China; ^2^ Shanghai Institute of Dermatology, Shanghai, China

**Keywords:** T-cell exhaustion, autoimmunity, immune-mediated inflammatory diseases(IMIDs), therapeutic exhaustion, inhibitory receptor, immunotherapy

## Abstract

Immune-mediated inflammatory diseases(IMIDs) are referred to as highly disabling chronic diseases affecting different organs and systems. Inappropriate or excessive immune responses with chronic inflammation are typical manifestations. Usually in patients with chronic infection and cancer, due to long-term exposure to persistent antigens and inflammation microenvironment, T-cells are continuously stimulated and gradually differentiate into an exhausted state. Exhausted T-cells gradually lose effector function and characteristics of memory T-cells. However, existing studies have found that exhausted T-cells are not only present in the infection and tumor environment, but also in autoimmunity, and are associated with better prognosis of IMIDs. This suggests new prospects for the application of this reversible process of T-cell exhaustion in the treatment of IMID. This review will focus on the research progress of T-cell exhaustion in several IMIDs and its potential application for diagnosis and treatment in IMIDs.

## Introduction

IMIDs are commonly autoimmune-mediated or suspected to be autoimmune diseases, which cause damage to the body’s own tissues as a result of a hyper-activated immune environment that is “misinduced” in the body and the body’s immune system response to its own antigens (1, 2). IMIDs can be triggered in a variety of ways, and symptoms vary depending on the diseases and the organs involved. How to suppress this state of overloaded immunity and regulate the immune balance of the body remains a critical issue.

T-cell exhaustion is a state of cellular dysfunction. It describes a late T-cell-differentiation state that actively inhibits cellular function (3). In cancer and infection, exhausted T-cells do not function fully and thus diminish the ability of the body’s immune system to defend itself against pathogens or tumor cells, resulting in an incomplete immune response that drives the spread of the pathogen or tumor cells (4). Based on extensive experiments, it was found that both humans and mice have exhausted T-cells which play a role in cancer (5) and many chronic infections (including human immunodeficiency virus, hepatitis B, and hepatitis C, etc.) (6).

**Figure 1:**
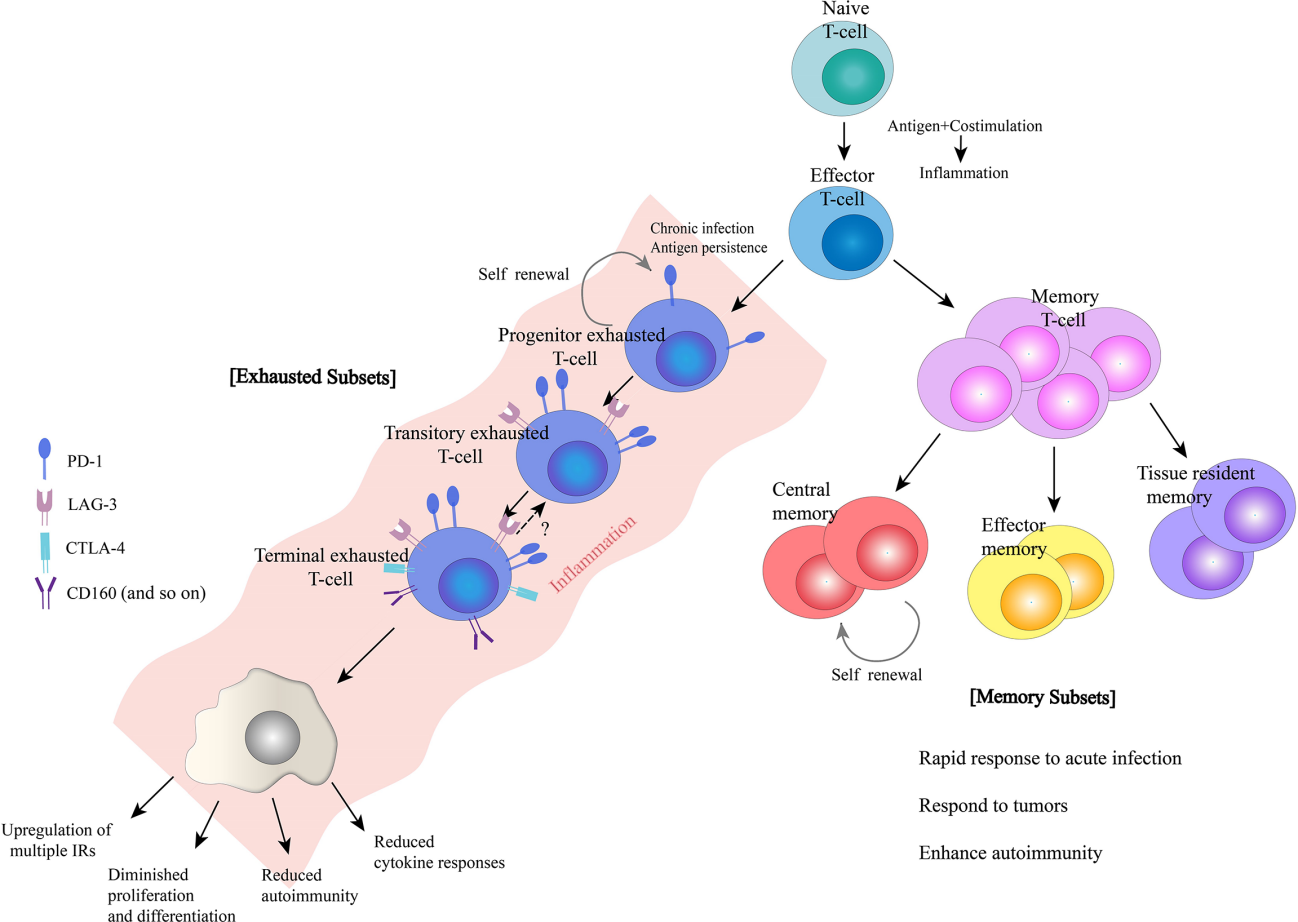
Characteristics of exhausted and memory T-cells. Naïve T-cells differentiate into effector T-cells, and then, a subset of effector T-cells differentiate into memory T-cells. In addition, in the presence of chronic infection and antigen persistence, T-cells may become exhausted. Both memory and exhausted T-cells have distinct subsets during differentiation that work together to perform their respective effector function.

Typical features of T-cell exhaustion include the progressive loss of effector functions with upregulation of multiple inhibitory receptors (IRs) such as programmed cell death protein1 (PD-1), T-cell immunoglobulin domain and mucin domain-3 (TIM-3), lymphocyte activation gene-3 (LAG-3), and T-cell immune receptor with Ig and ITIM domains (TIGIT), diminished proliferation and differentiation, diminished cytokine responses (especially interleukin [IL]-2, tumor necrosis factor-α, and interferon-γ), an altered cellular metabolic profile, an altered cellular gene-expression profile, and an altered epigenetic profile (4, 7). T-cell exhaustion is a continuous differentiation process in which T-cells are transformed from precursor cells to terminally exhausted cells. According to studies of T-cell exhaustion in cancer and chronic viral infections, the process of T-cell exhaustion can be broadly divided into three phases: sustained antigenic stimulation, negative costimulatory signaling, and chronic inflammation (6) (**Figure 1**).

To better characterize T-cell exhaustion, researchers compared T-cell differentiation after acute infection or vaccination with that in chronic infection or cancer states. Initially, driven by antigen recognition and transcriptional regulation, primitive naïve T-cells differentiate into short-lived effector T-cells and memory precursor effector T-cells, which produce cytokines and chemokines and acquire the ability to kill target cells (6, 8). After antigen clearance in the acutely infected or vaccinated organism, the majority of effector T-cells experience death, while a fraction of effector T- cells differentiate into long-lived memory T-cells, an action which is driven by a combination of antigen-stimulated production of IL-7, IL-15, IL-21 and the transcription factors T-bet and Eomes (9). In the case of chronic infection or cancer, although the initial process is similar, the differentiation state of T-cells gradually deviates from its normal course due to the persistence of antigenic stimulation. Because the precursor effector T-cells cannot completely clear the antigen, under continuous stimulation of the surviving antigen, the T-cells undergo a differentiation process of early exhaustion (10, 11). During that process, the T-cells steadily regulate their proliferation and effector functions to lower levels to cause minimal damage but still maintain some effector function to barely mediate a critical level of antigen-killing activity and tumor defense (12).With the persistence of antigens, T-cells in an early exhaustion state will further differentiate into a state of terminal exhaustion, and the effector function will be further suppressed or even completely deactivated (13). Currently, the clinical use of PD-1 antibodies and chimeric antigen receptor-modified T-cell (CAR-T) therapies is being pursued for the treatment of tumors and chronic infectious diseases (14). The desire to enhance the body’s immunity by reversing T-cell exhaustion has also revealed the superior ability of T-cells to kill tumor cells. However, most people who are treated, even those who initially respond well and show good treatment outcomes, can relapse despite the presence of T-cells in the body.

The precise mechanisms controlling the entry of T-cells into an exhausted state are still poorly understood. Although transcription factors, such as TOX and XBP1 and so on, are important regulators of T- cell exhaustion in persistent infection or cancer settings (15, 16), the existence of other key transcription factors remains controversial (**Table 1**). Beltra, J. C. et al. (39), based on transcriptional and epigenetic analysis, identified four developmental stages of exhausted T-cells and the control mechanisms of transitions between exhausted T-cell subsets. They also defined the key role of TCF-1, T-bet and Tox in the process, which refined the molecular, transcriptional and epigenetic mechanisms of T-cell exhaustion. In addition, the origin of environmental factors affecting T-cell exhaustion and the mechanisms by which they are generated to drive T-cell exhaustion remain unclear.

**Table 1:** Overview of transcriptional factors and E3 ligases involved in T-cell exhaustion.

Transcriptional factor	Role in exhausted T-cells	Reference
**TOX**	∙ Commit to program exhausted T-cells	
	∙ Regulate tumor-specific T-cell differentiation	(17–19)
	∙ Reinforce the phenotype and longevity of exhausted T-cells	
**NFAT**	∙ Promote T-cell exhaustion	(20, 21)
**NR4A**	∙ Promote T-cell exhaustion	(17)
**TCF-1**	∙ Amplify immunoreaction and improve the response to immunotherapy	(22, 23)
**IRF4**	∙ Promote T-cell exhaustion	(24)
	∙ Limit the development of Memory-like T-cells	
**IRF9**	∙ Limit early LCMV replication by regulating expression of interferon-stimulated genes	(25)
	∙ Prevent T-cell exhaustion	
**BATF**	∙ Maintain a permissive chromatin structure that allows the transition from TCF-1+ progenitors to CX3CR1+ effector cells	(26, 27)
	∙ Mediate the transition of T-cells away from exhaustion	
** **	∙ Skew progenitor T-cells toward effector phenotype	
**BLIMP1**	∙ Promote T-cell exhaustion	(28)
**T-bet**	∙ T-bet negatively regulates the transcription of Pdcd1 and thus T cell exhaustion	(29, 30)
	∙ High ratio of Eomes:T-bet in the nucleus of TEXs correlates with PD-1 expression and exhaustion	
**Eomes**	∙ Eomes expression positively correlates with high IRs expression and other features of more severe T cell exhaustion	(29, 31, 32)
	∙ High ratio of Eomes:T-bet in the nucleus of TEXs correlates with PD-1 expression and exhaustion	
**C-Jun**	∙ Downregulation/Functional deficiency in c-Jun mediates dysfunction in exhausted T-cells	(33, 34)
**E3 ligases**	**Role in exhausted T-cells**	**Reference**
** **	∙ Expression of Cbl-b is essential to virus-specific CD8+ T-cell exhaustion during chronic infection	(35, 36)
**Cbl-b**	∙ Deficiency of Cbl-b overcomes endogenous CD8+ T-cell exhaustion	
	∙ Deletion of Cbl-b in CAR T-cells renders them resistant to exhaustion	
**RNF183**	∙ Expression of RNF183 is significantly correlated with the expression of T-cell exhaustion markers	(37)
**Pellino1 (Peli1)**	∙ The development of Peli1-deficient CD8+ TILs prevented T-cell exhaustion and retained the hyperactivated states of T-cells to eliminate tumors	(38)

Cbl-b, casitas b-lineage lymphoma b; RNF-183, RING finger protein 183; TOX, thymocyte selection-associated high mobility group box; NFAT, nuclear factor of activated T-cells; NR4A, nuclear receptor subfamily 4 group A member; TCF-1, transcription factor T cell factor 1; IRF4, interferon regulatory factor 4; IRF9, interferon regulatory factor 9; BATF, basic leucine zipper transcription factor; BLIMP1, B lymphocyte induced maturation protein 1; T-bet, T-box expressed in T cell; Eomes, eomesodermin.

The defects of exhausted T-cells in defense against viral infection and tumor suppression have been well confirmed (14). In the tumor microenvironment, T-cell exhaustion accelerates the collapse of anti-tumor immunity and leads to the development of immune tolerance. However, there may be situations in which certain processes that suppress immunity are beneficial, such as in the case of organ transplantation or immune hyperactivation (40, 41). This area of application is less studied than tumor immunity or that in infectious diseases, but it has been shown that similar immunosuppressive processes can balance immune hyperactivation in the body and thus contribute to immune homeostasis (42). The transcriptional profile of T-cells is associated with the therapeutic response and prognosis of several immune-mediated and autoimmune diseases, especially systemic diseases like systemic lupus erythematosus (SLE), type 1 diabetes mellitus (T1DM), anti-neutrophil cytoplasmic antibody (ANCA)-associated vasculitis, rheumatoid arthritis (RA), and idiopathic pulmonary fibrosis (IPF) (42, 43), although the initial process is similar. Several studies have shown that a greater level of T-cell exhaustion is associated with a better prognosis of autoimmune diseases (3, 43). Therefore, we may conclude that T-cell exhaustion is a double-edged sword for immune homeostasis, playing a key role in tumor defense and infectious diseases as well as another one in autoimmune diseases to varying degrees (41). Although checkpoint blockers are currently widely used in clinical practice for immunotherapy of cancer (44, 45), their reverse process (i.e., the induction of checkpoint pathways to alleviate the autoimmune state) has not been as thoroughly explored, and targeted research in this area may provide new therapeutic opportunities. This review will provide an overview of the research progress on T-cell exhaustion in IMIDs.

## T-cell exhaustion in SLE/lupus nephritis

SLE is an immune-mediated and rheumatic autoimmune disease with systemic involvement. It is characterized by multi-organ inflammation and injury due to auto-antibody production, cell-tissue infiltration, immune tolerance, and a series of severe complications (46). T-cells promote the autoimmune response by assisting B-cells to activate antigen-presenting cells and release large amounts of cytokines.

It is considered that T-cells play a key role in lupus pathology (46). In addition, G. Lima et al. (47) suggested that T-cell exhaustion may also be a mechanism of immune tolerance in SLE patients. They compared the CD4^+^/CD8^+^ T-cell state in peripheral blood of two groups of SLE patients (including 15 patients in long-term remission who had never received treatment or had not received treatment for ≥ 10 years [Systemic Lupus Erythematosus Disease Activity Index 2000 score = 0 points] and 15 patients with active SLE [Systemic Lupus Erythematosus Disease Activity Index 2000 score ≥ 3 points], respectively) and a healthy population of 29 individuals by flow cytometry analysis, then defined seven CD4^+^ T-cell subpopulations and five CD8^+^ T-cell subpopulations in cases where the total number of CD4^+^ and CD8^+^ T-cells did not differ statistically. In comparison, the proportion of T-cell subsets expressing an exhaustion phenotype was significantly higher among patients in the long-term remission group, suggesting that T-cell exhaustion may serve as an important link in the immune tolerance of the organism in SLE patients.

However, most studies have focused on peripheral T-cells derived from blood or lymphoid tissue, and there is a lack of clear understanding of how tissue-specific T-cells function in SLE, where lupus nephritis is the most common and a very serious complication. Current research has begun to focus on the functional and phenotypic characteristics of kidney-infiltrating T-cells (KITs) derived from diseased kidney tissue in murine models of lupus (MRL/lpr, etc). Surprisingly, it was previously recognized that KITs are activated and expanded in tissue as the disease progresses and express a range of activated phenotypes. However, Jeremy S. Tilstra et al. (48) proposed that KITs do not express an activated effector T-cell profile and instead exhibit a series of attributes of T-cell function suppression, such as reduced cytokine production, low proliferation and differentiation capacity, and high expression of inhibitory receptors on the surface of T-cells, presenting a T-cell exhaustion-like profile. Their experiments confirmed significant suppression of the function of KITs in diseased kidneys by comparing the nature of KITs of MRL/lpr mice with that of peripheral spleen-derived T-cells and with the function and phenotype of KITs of C57BL/6 (B6) mice, which served as normal controls. This unexpected finding raises the question of how the “exhaustion” phenotype of KITs is reconciled with ongoing LN tisuee desruction. To address this question, they subsequently performed the detection of scRNA-sequencing and TCR-sequencing KITs in murine lupus model (49). They found that CD8^+^ KITs were first in a transitional state and then clonally expanded and evolved towards exhaustion. These transitional populations were first elucidated and may hold the key to understanding the dynamic processes of the tissue damage. On the other hand, CD4^+^ KITs do not fit into the current differentiation paradigms, but include hypoxia and cytotoxic subpopulations, with general exhaustion characteristics. And it has been reported that hypoxia can directly induce T-cell exhaustion (50). To explore the possible mechanisms involved, they found higher levels of messenger RNA for tumor necrosis factor-α and interferon-γ in MRL/lpr mouse CD8^+^ KITs than effector CD8^+^ T-cells in the spleen by transcriptional analysis, while the protein levels were reversed, suggesting that post-transcriptional regulation of cytokine genes (and possibly other related genes) may be one of the important links leading to the development of exhaustion, which needs to be demonstrated in subsequent experiments.

Although it has shown that the exhausiton populations of KITs are clearly present in murine lupus models (48), an exhaustion population was not observed in human LN in a report from the AMP consortium (51). In addition, Arnon Arazi et al. (52) compared KITs from normal and lupus nephritis patients by scRNA-sequencing and found that no significant difference in T-cell subsets between the groups (52), indicating that an exhausted T-cell subpopulation was not evident in KITs from lupus patients. This contradicts the results from the pathogenic murine models, suggesting the existence of a complex mechanism of lupus nephritis pathogenesis possibly due to the heterogeneity of KITs, and the local microenvironmental metabolism of tissues.

While these results suggest that it is theoretically possible that T-cell exhaustion may not occur in human LN and is mouse specific (48) and human tumor specific (53), it seems more likely that human autoimmunity has much in common with tumor environment. And thus it is reasonable to predict that with deeper research, exhausted KITs will be found in at least some patients. T-cell exhaustion is now clearly observed in peripheral blood CD8+ T-cells from lupus patients and is associated with a less propensity for disease progression (3, 52).

The above findings suggest a possible role of exhausted T-cells in the development of lupus, help to stratify the disease state, and offer new ideas for the study of disease heterogeneity. It provides a reliable basis for carrying out novel therapeutic strategies to develop the most effective individualized treatment plans for patients.

## T-cell exhaustion in rheumatoid arthritis

Rheumatoid arthritis (RA) is a common acute or chronic inflammatory immune disease of the connective tissue characterized by progressive joint inflammation leading to tissue damage. T-cells are the main effector cells (54, 55). Despite the presence of multiple pro-inflammatory cytokines and cells in the synovium, there is a component of the immune system that regulates the inflammatory response and maintains peripheral tolerance. The hyporeactivity of T-cells in the periphery and synovium of patients with RA was identified decades ago (56, 57). However, with the increasing research on T-cells in recent years, there have been new questions raised about whether T-cells in RA tissues are functioning in an ‘exhausted state’.

In fact, reduced T-cell function in RA synovial fluid mononuclear cells (SFMCs) was reported several years ago (58, 59). Based on these previous data, combined with the findings of Stinne R. Greisen et al. (60), high expression of co-inhibitory receptors on T-cells was confirmed in RA patients. RA joints can be considered as a microenvironment potentially favorable to T-cell exhaustion.

Amalia P. Raptopoulou et al. (61) previously found that PD-1/programmed death-ligand 1(PD-L1) expression was increased in both the synovial tissue and synovial fluid of RA patients. Notably, their immunohistochemical findings in synovial tissue suggested that the PD-1/PD-L1 pathway is likely to be involved in the pathological process of RA, which is reinforced by the finding of a greater susceptibility to collagen-induced arthritis (CIA) in PD-1^-/-^ mice. They also found that synovial fluid from RA patients was more enriched in PD-1^+^CD4^+^ T-cells than in peripheral blood, but there was no significant difference in PD-L1 expression between RA patients and normal controls. This result was confirmed in the study by Frederique M Moret et al. (62), suggesting an important role for the co-inhibitory PD-1/PD-L1 pathway in the regulation of inflammatory homeostasis in the inflammatory state of both humans and mice.

A similar point was demonstrated by Theresa Frenz et al. (63), who recorded significantly higher CD25 expression and expression of inhibitory receptors, PD-1 and CTLA-4, in CD4^+^ T-cells in peripheral blood and reduced expression of co-stimulatory factors like OX40, 41-BB, and CD69 in RA patients. By comparing normal controls and osteoarthritic spondyloarthritis patients, it was also found that IL-2 levels in peripheral blood were decreased in RA patients. This indicated that the CD4^+^ T-cells in RA patients show varying degrees of exhaustion. However, for CD8^+^ T-cells, although it has been found that the homeostasis of CD8^+^ T-cell in RA patients is indeed altered (64) and that the proportion of CD8^+^ T-cells negatively correlates with RA disease activity (64), little is known about their function and phenotype.

Membrane LAG-3, as one of the co-inhibitory receptors suggesting T-cell exhaustion, negatively regulates the proliferation, activation and function of CD4^+^ and CD8^+^ T-cells. Instead, the metalloproteases, ADAM10 and ADAM17 (65, 66), cleave the membrane form of LAG-3 at the connecting peptide site in the extracellular portion, leading to the formation of the monomeric soluble form of LAG-3(sLAG-3) (67). The study by Nayyereh Saadati et al. (68) indicated that serum level of sLAG-3 in RA patients was significantly higher than that of the healthy controls. Moreover, the serum level of sLAG-3 was different among the newly diagnosed patients without treatment, patients with active RA and patients in remission phase. However, there is no significant difference in the serum level of sLAG-3 between patients with moderate to severe disease activity and patients in remission phase.These differences may suggest the role of sLAG-3 in immune tolerance of RA. Although there is no direct evidence to show that the level of T-cell membrane LAG-3 changes in RA patients and its association with “exhaustion”, the complex relationship between membrane LAG-3 and sLAG-3 reflects the significance of the research to a certain extent.

The findings of Stinne R. Greisen et al. (69) focused on the fact that extracellular vesicles (EVs) are emerging as important transporters in the immune system, providing a protective pathway for the transport of genetic material and proteins between cells. Similar phenomena have been found in several other autoimmune diseases (69). These investigators pointed out the concept of intercellular transfer of receptors, where EVs in RA patients transfer their transporters (possibly microRNAs) to cells in the microenvironment, involving the development of T-cell exhaustion and the transfer of the co-suppressor receptor PD-1, thus increasing the chronic development of these patients. EVs could become new therapeutic targets as a result.

These findings provide us with new therapeutic ideas to balance the ‘pathogenic’ T-cells in RA. However, there is no definite finding to clarify the exhausted phenotype and functional status of T-cells present in RA patients, but this is quite valuable as a future direction to explore.

## T-cell exhaustion in type I diabetes mellitus

T1DM is an organ-specific IMID that leads to the destruction of pancreatic islet β-cells, resulting in dysregulation of blood glucose and lifelong dependence on exogenous insulin therapy. In mouse models, CD8^+^ T-cells have recently emerged as crucial factors in the pathogenesis of T1DM (70). Notably, islet-specific CD8^+^ T-cells can be detected in the peripheral blood of T1DM patients (71). The disease process of T1DM is mainly mediated by this group of cells. When the body’s tolerance system relapses, islet β-cells are destroyed by immune cells, so existing therapeutic studies focus on enhancing autoimmune tolerance to prevent relapse, in addition to depleting or suppressing autoimmune-active cells (72).

In a cross-sectional study of T1DM patients (73, 74), those identified as having slow disease progression (classified by serum C-peptide level) had an increased proportion of specific CD8^+^T-cells expressing “exhaustion” phenotype. And these exhausted CD8^+^T-cells expressed high level of chemokine receptor CXCR3 and transcription factor EOMES. However, these changes were not significantly correlated with disease duration and age in T1DM patients. The study also indicates that increasing exhausted islet-specific CD8^+^T-cells can slow down the progression of β-cell loss caused by autoimmune disruption.

The study by Avanzini, M.A. et al. (75)found that the level of IFN-γ produced by peripheral blood CD4^+^ and CD8^+^T-cell was significantly lower in T1DM patients than in healthy controls and patients at risk. The 15-month follow-up patients also showed significantly lower level of IFN-γ produced by peripheral blood CD4^+^ and CD8^+^ T-cells compared with other groups. Moreover, according to the 8-year follow-up, no significant differences were observed among the groups. These T-cell subsets could have been considered as “exhausted cells”.

Exhausted CD8^+^ T-cells were also found to be correlated with therapeutic response in subjects using an anti-CD3 antibody (teplizumab) in relevant immunotherapy clinical trials (76, 77). S. Alice Long et al. (76) found that islet-specific CD8^+^ T-cells from healthy individuals and T1DM patients exhibited heterogeneous phenotypes. The disease-progression rates in T1DM subjects were associated with two shared phenotypes: the activated and exhausted phenotypes. The former was more common in the rapid-progression group, whereas the latter was more common in the slow-progression group. Peter S. Linsley et al. (78) also found that islet-specific CD8^+^ T-cell phenotypes often included ≥ 1 distinct phenotype and subjects with a greater proportion of exhausted islet-specific CD8^+^ T-cells exhibited experienced slower disease progression in T1DM. These exhausted CD8^+^ T-cells recognize auto-antigens extensively and proliferate *in vitro* at low levels, and ligand agonists of the inhibitory receptor further reduce the activity of such exhausted cells, suggesting that their exhausted phenotype may not be the final differentiation state (76). This data suggest that the phenotype and function of autoreactive CD8^+^ T-cells are key factors in disease progression.

There are currently two potential directions for T1DM that target the treatment of T-cell exhaustion states. The first is to induce T-cell exhaustion in individuals who still exhibit an ‘unexhausted’ phenotype, thereby achieving a delay in the disease progression of T1DM (43, 74). Creating an environment of chronic antigen exposure may be one way to achieve this. The second approach is to activate inhibitory receptors expressed by the exhausted T-cells because the exhausted T-cells are not completely deactivated and maintain some immune activity (79). Although the induction of CD8^+^ T-cell exhaustion to mitigate autoimmune destruction of islet β-cells is a highly regarded therapeutic pathway, even if it does not permanently block disease progression in T1DM, it has shown promising effects in mitigating islet β-cell loss (74). In conclusion, induction of an enhanced and expanded T-cell exhaustion state may be a beneficial therapeutic pathway for T1DM.

## T-cell exhaustion in other IMIDs

An increasing amount of experimental data demonstrate that T-cell exhaustion in autoimmune diseases can limit the activation state of T-cells and is associated with a good prognosis of the disease.

ANCA-associated vasculitis (AAV) is a group of systemic small-vessel vasculitis conditions most notably characterized by detectable ANCA concentrations in the serum. Abnormal CD8^+^ T-cells are associated with the development of AAV (80). Based on a cohort study of 59 patients diagnosed with AAV, Eoin F McKinney et al. (3) found that gene-expression profiles reflecting CD8^+^ T-cell exhaustion were similar to those found in chronic viral infections, and were associated with a low relapse rate in patients with AAV during follow-up. These investigators sought to construct a predictive model to explain the correlation between the phenotypic characteristics of CD8^+^ T-cells and the clinical disease state, and possibly as a biomarker for predicting disease prognosis (3).

Several studies have identified a similar phenomenon in the experimental autoimmune encephalomyelitis (EAE) mouse model. EAE is an autoimmune disease primarily driven by the reactivity of inflammatory T-cells to myelin antigens. Manu Rangachari et al. (81) based on the experimental conjecture that Bat3 can protect Th1 cells from the inhibitory function of TIM-3, performed a Bat3-knockout experiment in an EAE mouse model and noted that Bat3 deletion significantly reduces the frequency of cells producing interferon-γ and IL-2, accompanied by an increased expression of TIM-3 and greater production of IL-10. This phenotype is reminiscent of the state of T-cell exhaustion. It has also been shown in many previous studies that TIM-3 is an important inhibitory receptor that indicates a state of T-cell exhaustion (82, 83). Such a series of critical T-cell phenotypes suggest a correlation between T-cell exhaustion and EAE disease progression.

Multiple sclerosis (MS) is considered an autoimmune disease of the central nervous system in which immune responses mediated by autoreactive T-cells appear to play a critical role (84). In 95 patients and 56 Epstein–Barr virus (EBV)-seropositive healthy subjects, Michael P Pender et al. (85) confirmed that CD8^+^ T-cell responses to EBV lysis but not cytomegalovirus lysis antigens were found to be reduced during periods of multiple sclerosis flare and in all subsequent disease stages of patients. CD8^+^ T-cells targeting EBV latent antigens were increased but cytokine multi-functionality was decreased, indicating a state of T-cell exhaustion. During viral challenge, EBV-specific CD4^+^ and CD8^+^ T-cell populations expand and latent-specific CD8^+^ T-cell function increases. There was a progressive decrease in EBV-specific CD4^+^ and CD8^+^ T-cells with increasing disease duration, consistent with T-cell exhaustion.

Similar T-cell exhaustion also occurs during transplant tolerance (86). Several studies have demonstrated that some of the typical inhibitory pathways in T-cell exhaustion, such as CTLA-4, TIM-3, PD-1/PD-L1/2, also play a role in transplantation tolerance (87, 88). The role of T-cell exhaustion in the prevention of allogeneic rejection and the induction of tolerance is also gaining recognition. Further understanding of the mechanisms of induction of T-cell exhaustion is necessary to promote treatment strategies.

## Exhausted T-cells or dysfunctional T-cells?

T-cells play a key role in clearing pathogens and controlling tumor progression (89). However, during chronic infections and tumor development, persistent antigenic and inflammatory stimuli can cause T-cells to enter a state of exhaustion. Exhausted T-cells are a unique cell lineage characterized by a progressive loss of effector functions; sustained high expression of inhibitory receptors; dysregulations in cellular metabolism, memory function and the capacity for self-proliferative differentiation; and altered transcriptional and epigenetic profiles, gene-expression profiles (6).

However, a consensus on the definition of T-cell exhaustion has not yet been reached. Therefore, it was concluded that exhausted T-cells are dysfunctional, but dysfunctional T-cells are not necessarily exhausted T-cells. In other words, the dysfunctional and exhausted states of T-cells cannot be classified as identical concepts (90, 91). The definition of exhausted T-cells therefore needs to rely on not only the assessment of T-cell phenotype and function state but also the cell-differentiation state through a combination of molecular and transcriptional profiles. Genomic approaches have now been applied in studies to progressively identify the main features of T-cell exhaustion, the role of inhibitory receptors, and changes in the downstream signaling pathways and cellular metabolic profiles of the T-cell receptor (TCR) and related cytokines (92). As a result, it provides evidence for whether T-cell exhaustion can act as a distinct cell lineage.

Although current research on T-cell exhaustion has focused on CD8^+^ T-cells, the impact of exhaustion states of other immune cell types, including CD4^+^ T-cells, in cancer and other disease states remains relatively underestimated. However, given the important role of CD4^+^ T-cells and other immune cell types in the immune response, we need to clarify the phenotypic and functional similarities as well as the differential associations between CD4^+^ T-cell or other immune cell exhaustion and CD8^+^ T-cell exhaustion, respectively, and to discern how these cells interact with CD8^+^ T-cells to cause their exhaustion state. Current research on CD4^+^ T-cell exhaustion has focused on phenotypical studies. Several scholars have found that CD4^+^ T-cells in chronic infection and recurrent infections exhibit upregulation of inhibitory receptors that typically mimic CD8^+^ T-cell exhaustion (93–95). And increased co-inhibitory receptor expression is found to be associated with more advanced disease states and decreased progression-free survival (96–98). In addition, in the presence of persistent antigens, such CD4^+^ T-cells have also been found to exhibit reduced cytokine production, antigen-specific immune responses and weakened secondary immune responses (99, 100). Upregulation of inhibitory receptors associated with exhaustion have also been identified in various tumor models in human and mice (101–103) and transplantation (104). Beyond that it is necessary to continue to explore the metabolic profiling, epigenetic landscape, etc of CD4^+^ T-cells in different disease states to determine the exhaustion state of CD4^+^ T-cells, and whether there is also a unique differentiation state of CD4^+^ T-cell exhaustion. This is critical for us to understand the mechanism of T-cell exhaustion and its therapeutic implications.

Adrien Leite Pereira et al. (105), after examining the multidimensional phenotypic characteristics of various types of immune cells in RA patients, proposed that all types of immune cell subpopulations may haven an exhaustion phenotype, and they expect to continue to explore the relationship of the exhaustion phenotype of T-cells with disease activity. Kirsten Richter et al. (106) also suggested that, regardless of the type of antigen-presenting cells, it is the amount of antigen that is continuously stimulated that is the key determinant of T-cell exhaustion during chronic viral infection.

Defining whether an exhaustion phenotype also exists in various cell subpopulations and discerning how it affects the immune response and disease severity are critical to our understanding of immune- regulatory processes in the pathology of various diseases and knowing whether we can specifically target different cells for new therapeutic studies.

## A positive side to T-cell exhaustion

The main body of research on T-cell exhaustion remains focused on chronic infections and cancer, in which it plays a negative role. However, there are also situations in which the body needs to suppress immunity activity, such as autoimmune diseases and transplantation. It has been proposed that the transcriptional profile of T-cells correlates with the prognosis of several autoimmune diseases (including SLE, T1DM, ANCA-associated vasculitis, Idiopathic pulmonary fibrosis, and dengue hemorrhagic fever) (107). This also supports the idea that T-cell exhaustion does not only have negative effects on the body but also plays a key role in the outcome of infectious diseases, cancer, and autoimmune diseases.

## Translating T-cell exhaustion from chronic infection and cancer to IMIDs: Inhibitory receptors in T-cell exhaustion and their implications for targeted prognosis and treatment

Exhausted T-cells overexpress inhibitory receptors, including PD-1, LAG-3, TIM-3, 2B4/CD244, CD160, TIGIT, and other receptors that play a major role in regulating T-cells. Based on many studies, it is possible to offer a new concept that T-cell exhaustion is reversible and that it is possible to activate exhausted T-cell responses and enhance their control of viral infections and tumors by blocking inhibitory receptors *in vivo*. Anti-PD-1 therapy has become the hottest research direction and has already achieved a significant clinical trial effect (108).

Inhibitory and co-stimulatory receptors play a critical role in adaptive cellular immunity (109). Inhibitory receptors are the key negative regulatory pathways that control the body’s auto-reactivity and immunopathology (110), which play an indispensable role in maintaining immune homeostasis. The persistent high expression of inhibitory receptors is one of the major features of T-cell exhaustion, but we need to remain clear about the fact that T-cells expressing inhibitory receptors are not defined as exhausted T-cells. Expression of inhibitory receptors is not exclusive to exhausted T-cells, and many T-cell subpopulations in a highly activated state also express inhibitory receptors, and transiently express on effector T-cells. The PD-1/PD-L1/2 inhibitory pathway is one of the most studied inhibitory receptor pathways in T-cell exhaustion research to date. The PD-1 signaling pathway is known to play a role in negative immune regulation in a variety of infectious diseases and cancers, and its inhibitors are approved by the U.S. Food and Drug Administration for clinical treatment. It can be seen that the PD-1 pathway and reversal of T-cell exhaustion may have implications for the treatment of many diseases (111).

However, many questions remain unanswered, including those regarding the optimal drug dose to be used, drug safety, drug persistence, and exact drug efficacy. Also, little existing research has focused on the molecular mechanism of T-cell exhaustion manipulated by inhibitory receptors, and the network of T-cells regulated by inhibitory receptors, such as PD-1, is perhaps very complex, with the involvement of multiple mechanisms (111). Besides, PD-1 and its ligands PD-L1/2 are widely expressed in non-lymphoid tissues, and activation of the PD-1 pathway also mediates negative regulation of PI3K/PKA and Ras signaling pathways, which in turn inhibits T-cell receptor-mediated lymphocyte proliferation and effector cytokine production, implying a multitude of possible side effects.

**Figure 2:**
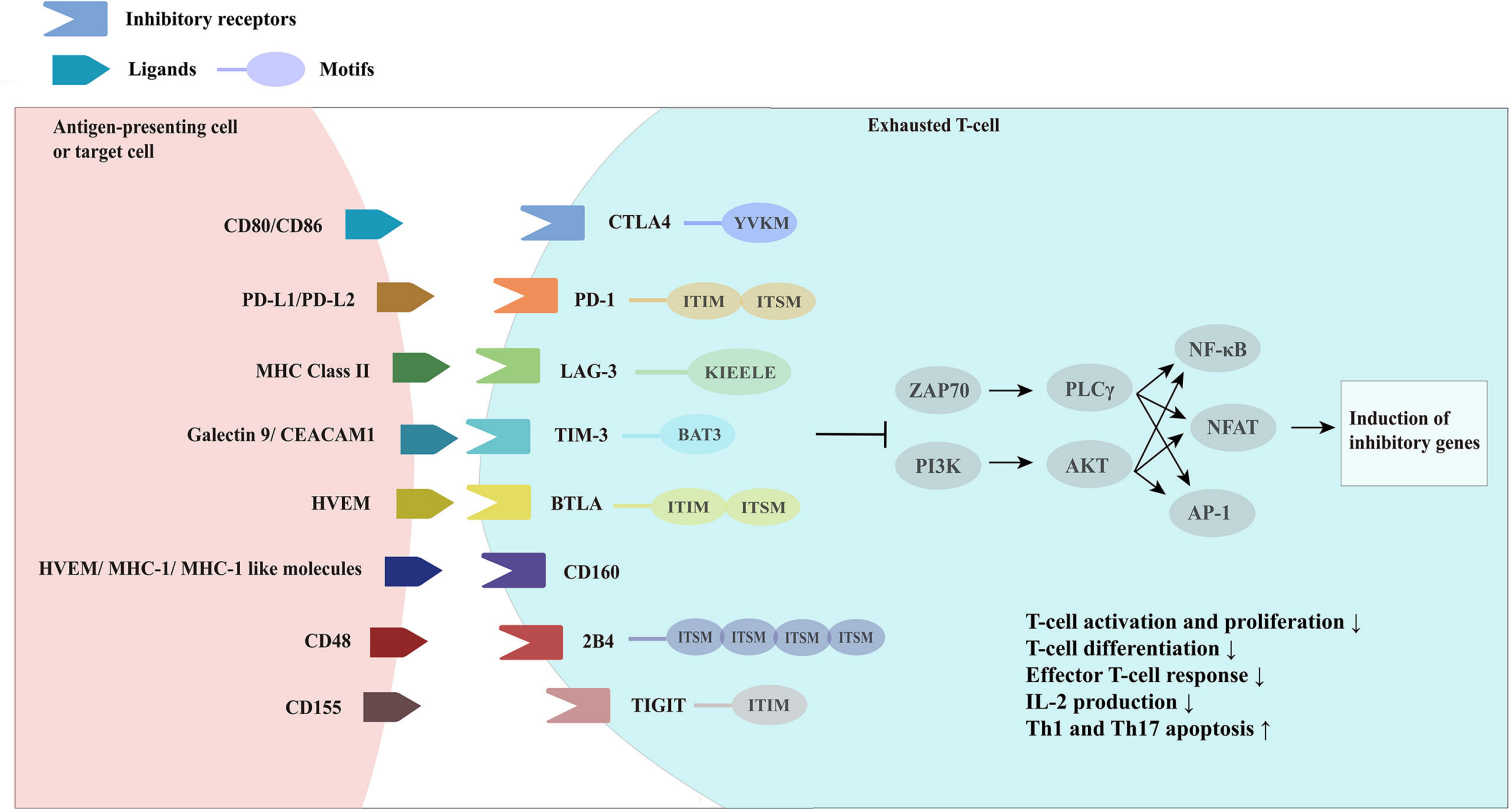
Overview of inhibitory receptors and related molecules in exhausted Tcells. CTLA4, cytotoxic T-lymphocyte associated protein four; PD-1, programmed cell death one; LAG-3, lymphocyte activation gene-3; MHC Class II, major histocompatibility complex class II; TIM-3, T-cell immunoglobin and mucin domain-3; CEACAM1, carcinoembryonic antigen-related cell adhesion molecule 1, BTLA, B-lymphocyte and Tlymphocyte attenuator; HVEM, herpes virus-entry mediator; 2B4, recombinant natural killer cell receptor 2B4; TIGIT, T-cell immunoreceptor with Ig and ITIM domains.

In addition to PD-1, exhausted T-cells also express a range of other inhibitory receptors, with PD-1 co-expressed with multiple inhibitory receptors, including LAG-3, TIM-3, CTLA4, and 2B4/CD244 (31) (**Figure 2**).

It was found that the greater the number of inhibitory receptors co-expressed by exhausted T-cells, the more severe the exhaustion state of these T-cells was (31). Although the expression of inhibitory receptors does not necessarily indicate that T-cells are in a state of exhaustion, inhibitory receptors are often misinterpreted as a marker of “exhaustion”, an association that is not reliably documented. While some studies have shown a clear association between the high expression of inhibitory receptors on T-cells and reduced cytokine release potential in the setting of chronic antigen exposure (viral infection or cancer), data from Amandine Legat et al. (112) suggest the opposite, indicating that environmental factors that stimulate T-cell activation and differentiation are actually the main triggers of inhibitory receptor expression (112). However, T-cells do function at some levels of suppressive dysregulation, and co-expression of multiple inhibitory receptors is a key feature in T-cell exhaustion. Thus, studies have identified the efficacy of combined blockade of inhibitory receptor therapies, which are derived from different structural receptor families, mediate different expression patterns and different properties of ligands and their downstream signaling pathways, and may have great potential for reversal of T-cell exhaustion therapies through appropriate combination-therapy (113).

It has also been proposed that patients with a good prognosis for each disease have their own unique set of upregulated exhaustion-associated inhibitory receptor subsets (3). During chronic LCMV infection in mice, T-cell exhaustion is driven by the concerted upregulation of multiple co-inhibitory receptors that synergistically signal, producing a state of systemic immunosuppression. In autoimmunity, these receptors are not coordinately upregulated as a population. The importance of T -cell exhaustion signaling in driving relapse or determining relapse risk and prognosis, rather than in disease susceptibility diagnosis or predicting disease activity, is emphasized. Perhaps by detecting the expression of these inhibitory receptors, they can be used as indicators of disease risk and prognosis. Another new insight emerge from this was that disease activity and recurrence are controlled by separate processes, and targeted control of these processes may lead to new treatment options.

Appropriate biomarkers that can stratify disease severity or predict disease prognosis enable therapies to be individualized for patients with autoimmune diseases and minimize the potential adverse effects of treatment. Gene expression-based biomarkers are already being used as predictors and monitors in cancer treatment (114, 115). Eoin F McKinney et al. (107) proposed a predictive model, adopting the transcriptional profile of CD8^+^ T-cells to explain their relevance to autoimmune diseases. We therefore also considered whether suitable predictive models of T-cell-exhaustion phenotypes exist to assess the severity stratification, prognosis, and therapeutic efficacy of autoimmune diseases.

## Therapeutic potential of T-cell exhaustion in autoimmunity: Therapeutic exhaustion

**Figure 3:**
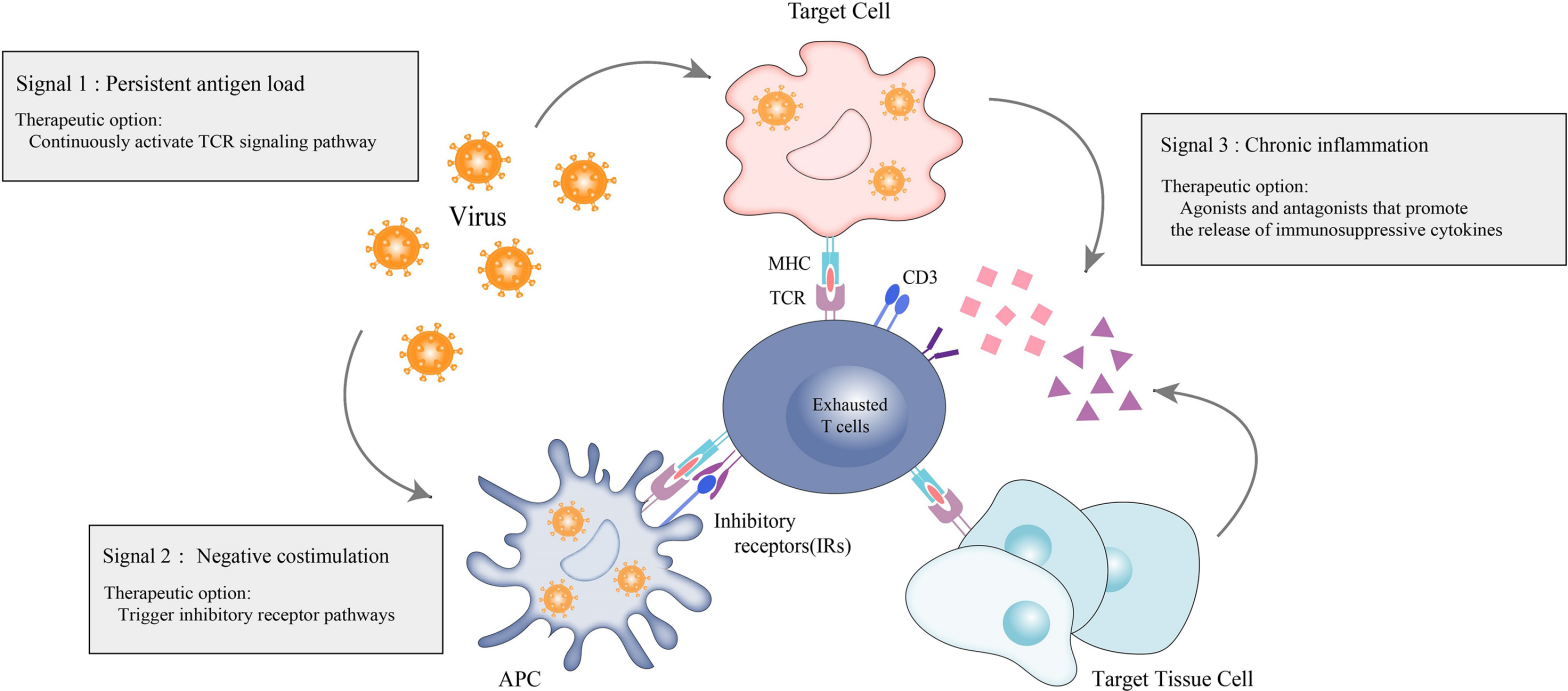
Therapeutic options for exhausted T-cells in autoimmunity. Therapeutic options for autoimmune diseases based on the hypothesis that promoting T-cell exhaustion can be therapeutic. Persistent antigen load from virus or tumor (signal 1) drives hyperactivation of T-cells and ultimately leads to sustained coexpression of multiple inhibitory receptors on T-cells and their ligands on antigen presenting cells (APCs), virally-infected cells and target tissue cells. The inhibitory receptors prompt negative costimulation (signal 2) to T-cells. In response to persistent antigen load, virally-infected cells, APCs and target tissue cells further drive exhaustion by producing proinflammatory cytokines and immunosuppressive cytokines to promote the exhaustion state of T-cells.

Patients with IMIDs have immune hyperactivation in their bodies, and the prevailing treatment strategy is broad immunosuppression to suppress excessive immune activation. However, this approach may increase the patient’s susceptibility to other common infections alongside the primary cause of their condition. L.M.McLane et al. (6) proposed a “three-signaling model” of T-cell exhaustion. Inspired by this, one of the most effective approaches is to continuously activate the TCR signaling pathway in the absence of co-stimulation to simulate an environment of persistent antigenic load. Other options include the enhancement of negative co-stimulation (e.g., triggering inhibitory receptor pathways) and the use of agonists or antagonists that promote the release of immunosuppressive cytokines, simulating the creation of a chronic inflammatory state (**Figure 3**). Such actions are complex, but, in general, can be chosen to promote a state of T-cell exhaustion in the organism in the opposite direction of reversing T-cell exhaustion, thus converging on immune balance of the organism, which is currently the dominant direction of research in T-cell exhaustion therapies in autoimmune diseases.

The concept of “therapeutic exhaustion” has been proposed as referring to the possibility of inducing some degree of T-cell exhaustion to treat autoimmune diseases. A major advantage of therapeutic exhaustion over systemic immunosuppression is that the artificial immunosuppression produced by induced exhaustion is antigen-specific, suggesting that we may have the opportunity to selectively suppress the immune response without compromising the body’s beneficial immune response against foreign antigens.

McKinney, E. F. et al. (3, 43) showed that the transcriptional signature of T-cell exhaustion is associated with self-protection during relapse in a variety of autoimmune diseases by constructing a model of *in vitro* co-stimulation of human primary CD8^+^ T-cells in which the CD8^+^ T-cells were activated in the presence or absence of antibody-mediated CD2 stimulation (using beaded polyclonal anti-CD3/28 antibodies). The addition of Fc-chimeric PD-L1 to this *in vitro* model was found to limit the differentiation of IL-7R^hi^ PD-1 ^lo^ cell populations, implying that checkpoint agonists may be able to shift T-cells toward an exhaustion phenotype. However, extensive *in vivo* experiments and clinical trials are still needed to observe the potential of this approach.

The therapeutic induction of autoimmune activation of T-cell exhaustion in the organism seems to be a logical option for immunotherapy. However, many potential issues still need to be discussed. First, therapeutic exhaustion currently targets inhibitory receptors on the T-cells’ surface. In other words, there is a clear target, and the applied checkpoint agonists should target inhibitory receptors on the surface of strongly co-stimulated memory CD8^+^ T-cells. However, the expression of such CD8^+^ T-cell inhibitory receptors on the surface of T-cells is low. Moreover, the previous study observed that a unique subset of inhibitory receptors highly expressed on the surfaces of CD8^+^ T-cells in different disease states presents disease specificity (22). Therefore, it is essential to employ appropriate checkpoint agonists or agonist combinations for different disease states to achieve ‘therapeutic exhaustion’.

In addition, the time of therapeutic exhaustion therapy administration is critical. Early in the course of the disease, increased expression of T-cell surface inhibitory receptors may be a mechanism by which the body protects itself from immune homeostasis, rather than entering a state of exhaustion, which results from sustained, rather than transient, long-term stimulation. Long-term support may be required to maintain the efficacy of therapeutic exhaustion. Moreover, as mentioned in the previous section, CD8^+^ T-cell exhaustion is a continuous differentiation process. Even when antigen levels are reduced or completely cleared, the exhaustion-associated T-cell phenotypic features are maintained through stable epigenetic modifications. At this point, the question of whether therapies for therapeutic exhaustion can alter such a state of “exhaustion homeostasis” remains to be explored.

As a novel therapy, we must consider the adverse effects that may result from induced exhaustion. The target site for induced exhaustion should be clear. However, inhibitory receptors are expressed on the surface of multiple immune cells, and there is a potential to trigger immune perturbation. Current investigations of therapies for reversing T-cell exhaustion have shown that combinations of different checkpoint blockers produce better efficacy results and fewer adverse effects. In the same way as therapeutic exhaustion, perhaps the application of combined checkpoint agonists could produce better effects and minimize adverse ones.

Therapeutic exhaustion has also been proposed as a means of prophylactic treatment, which requires us to identify early patients who may benefit from this therapy. In conclusion, the ultimate goal of therapeutic exhaustion is to modify the long-term biological process of autoimmune patients. However, a large number of studies are still needed to investigate its potential biological effects, and we should find appropriate biomarkers for its evaluation.

Importantly, this therapeutic concept is very abstract and has serious limitations in terms of research and future clinical implementation. Current research data on therapeutic exhaustion is very limited and not sufficient to demonstrate that a state of immune balance exists that can be achieved with “therapeutic exhaustion”. A large proportion of current therapies for autoimmune disease exhaustion have been translated from related cancer research. Thus, a fundamental problem is revealed in that most cancer immunotherapies based on T-cell exhaustion take the form of antagonizing inhibitory receptors expressed on the surface of exhausted T-cells, whereas, in autoimmune diseases. T-cell exhaustion appears to be negatively correlated with disease severity, and patients who benefit from exhaustion therapy have lower levels of inhibitory receptors expressed on T-cells (43).Therefore further studies are required to explore the intermediate links and potential molecular mechanisms affecting T-cell exhaustion.

## Limitations of T-cell exhaustion therapies

Although T-cell exhaustion is a process of continuous differentiation, exhaustion-associated phenotypic feathures are maintained through stable epigenetic modifications, even when antigen levels are reduced or completely cleared (13, 116),. Even with PD-1 blockade therapy, these stable epigenetics limit T-cell expansion and clonal diversity (117). In addition to epigenetic inheritance, the specific transcription factors TOX and NR4A have been identified as key regulators that drive and maintain the T-cell exhaustion state (17, 118). In the absence of TOX, no exhausted T-cells are produced. TOX drives T-cell exhaustion by mediating T-cell transcription and epigenetics. This poses a major problem for T-cell exhaustion therapies, with the possibility for disease relapse once drugs or treatments are withdrawn.

## Conclusion

Although significant progress has been made in recent years in the study of T-cell exhaustion, much research is still needed on how to regulate T-cell exhaustion and how best to reverse or promote the development of this state. In addition, most studies on T-cell exhaustion are based on mouse models. However, given the limitations of mouse models, such as the degree of immune cell infiltration and responsiveness to therapeutic interventions that differ from those in humans, their true clinical value is questionable, and we still do not know much about T-cell exhaustion in the microenvironment of the human disease state. Combining phenotype studies and functional studies is important. Future research is needed to explore the mechanisms and conduct relevant clinical studies.

## Author contributions

All authors listed have made a substantial, direct and intellectual contribution to the work, and approved it for publication.

## Conflict of interest

The authors declare that the research was conducted in the absence of any commercial or financial relationships that could be construed as a potential conflict of interest.

## Publisher’s note

All claims expressed in this article are solely those of the authors and do not necessarily represent those of their affiliated organizations, or those of the publisher, the editors and the reviewers. Any product that may be evaluated in this article, or claim that may be made by its manufacturer, is not guaranteed or endorsed by the publisher.
